# Wavefront-aberration-tolerant diffractive deep neural networks using volume holographic optical elements

**DOI:** 10.1038/s41598-024-82791-z

**Published:** 2025-01-07

**Authors:** Ikuo Hoshi, Koki Wakunami, Yasuyuki Ichihashi, Ryutaro Oi

**Affiliations:** Applied Electromagnetic Research Center, National Institute of Information and Communications Technology, Nukui-Kitamachi, Koganei, Tokyo 184-8795 Japan

**Keywords:** Diffractive deep neural network, Holographic optical element, Machine learning, Adaptive optics, Applied optics, Optical techniques

## Abstract

As the demand for computational performance in artificial intelligence (AI) continues to increase, diffractive deep neural networks (D^2^NNs), which can perform AI computing at the speed of light by repeated optical modulation with diffractive optical elements (DOEs), are attracting attention. DOEs are varied in terms of fabrication methods and materials, and among them, volume holographic optical elements (vHOEs) have unique features such as high selectivity and multiplex recordability for wavelength and angle. However, when those are used for D^2^NNs, they suffer from unknown wavefront aberrations compounded by multiple fabrication errors. Here, we propose a training method to adapt the model to be unknown wavefront aberrations and demonstrate a D^2^NN using vHOEs. As a result, the proposed method improved the classification accuracy by approximately 58 percentage points in the optical experiment, with the model trained to classify handwritten digits. The achievement of this study can be extended to the D^2^NN that enables the independent modulation of multiple wavelengths owing to their wavelength selectivity and wavelength division multiplex recordability. Therefore, it might be promising for various applications that require multiple wavelengths in parallel optical computing, bioimaging, and optical communication.

## Introduction

Deep neural networks (DNNs) are mathematical models that imitate the neurons of the human brain^1,2^ and have shown outstanding performance in various fields, such as voice recognition^3^, image classification^4^, image segmentation^5^, and computational imaging^6^. Moreover, much of what is called artificial intelligence (AI) has been based on DNNs in recent years. The remarkable development of DNNs has been supported by various domain-specific processors such as graphics processing units (GPUs)^4^ and field-programmable gate arrays^7^ as well as improvements in processor performance owing to the miniaturization and integration of semiconductors. However, processor performance improvements are reaching their limits despite the ever-increasing demands on the computational performance of DNNs. To address these issues, optical DNNs^8–13^, which use light instead of electrons for computation, are attracting attention since they enable high-speed computation with extremely low power consumption and low latency. Among optical DNNs, a diffractive deep neural network (D^2^NN)^14–16^ can perform DNN computing by repeating wavefront propagation and optical modulation. The amount of optical modulation at each layer is determined by *in silico* training in a computer simulation or *in situ* training optically^17^, and the desired optical output is realized using spatial light modulators (SLMs) or diffractive optical elements (DOEs). The greater the number of layers of DOEs, approximately up to 5 layers, and the higher the nonlinearity of the network, the more complex the functions that can be computed and the higher the performance that is achieved^14^. D^2^NNs are capable of wireless, extremely low power consumption, as well as low-latency and high-throughput computing through parallel computing utilizing spatial parallelism. Owing to these features, they can accelerate computation and perform distinctive tasks such as object segmentation^18^, three-dimensional object recognition^19^, logic operations^20^, beam shaping^21^, wavefront sensing^22^, adaptive optics^23^, and Gaussian beam classification^24^ by incorporating optical devices as DOEs.

**Fig. 1:**
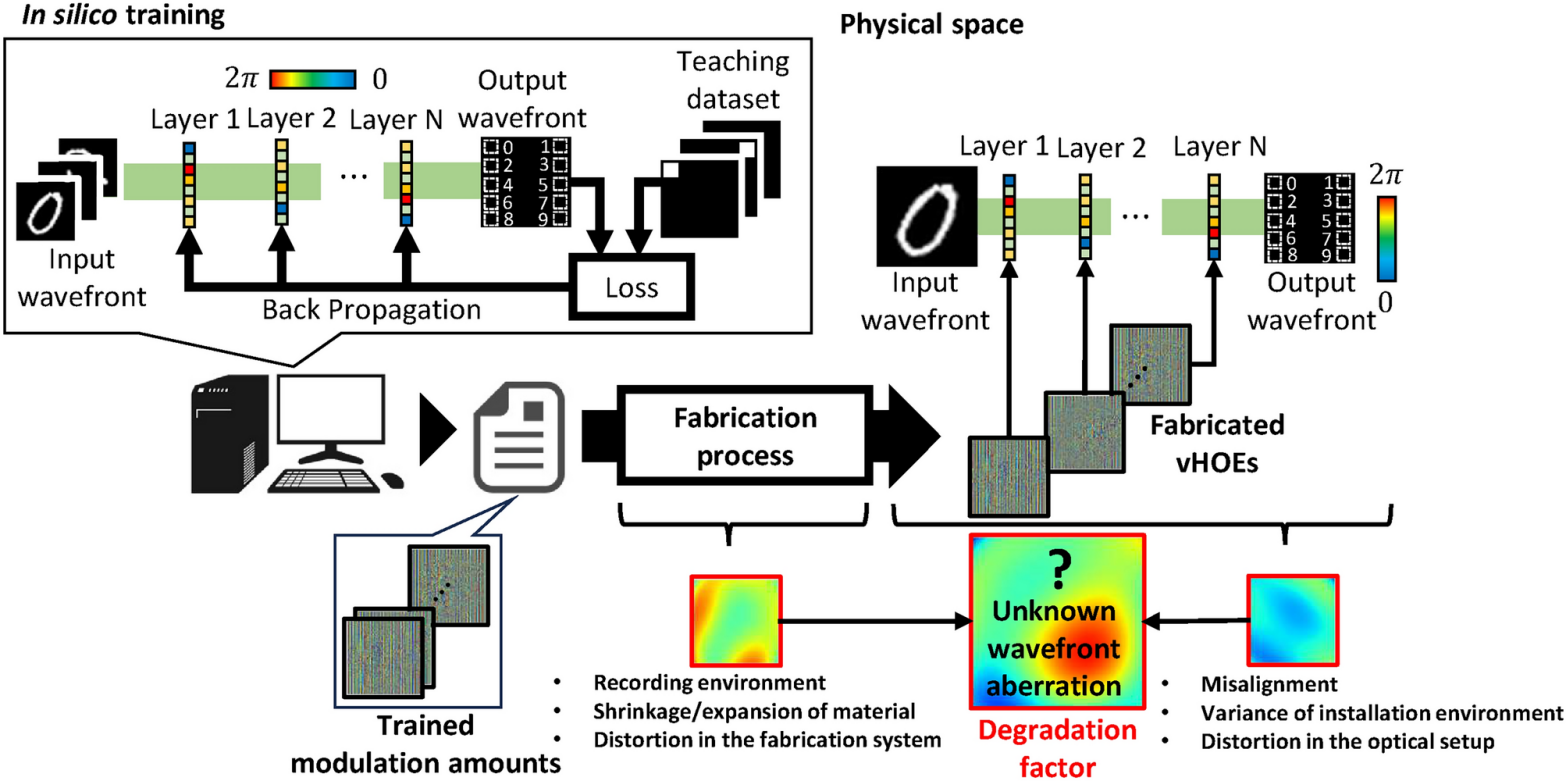
Process from in silico training to D^2^NN configuration using vHOEs in physical space.

DOEs used for D^2^NN implementation are varied in terms of fabrication methods and materials, such as 3D printers^14^, photolithography^15^, two-photon nanolithography^22,25^, magnetic materials^26^, and metasurfaces^27^. Among them, volume holographic optical elements (vHOEs), which are based on the principle of holography^28,29^, have unique characteristics such as high selectivity and division multiplex recordability for wavelength and angle. These features make it possible to realize, for example, the input of a wide range of incident angles and independent modulation for multiple wavelengths. Particularly for wavelength, many previous studies on, for example, multispectral snapshots^30,31^, classification tasks^32^, and wavelength parallel linear transformations^33^, have been reported. Many of these studies have led to the achievement of wavelength parallelism through creative learning or input/output designs. Therefore, wavelength parallelism owing to materials leads to the further development of wavelength parallelism in these fields. Figure 1 shows the flow from training to the implementation using vHOEs in a D^2^NN. At first, the amount of modulation is trained *in silico* since vHOEs are static elements. (See the Section “In silico training” for details of *in silico* training.) Then, vHOEs fabricated on the basis of the trained modulation amounts form the D^2^NN in physical space. However, the D^2^NN with vHOEs faces challenges in that vHOEs have been reported to shrink/expand with temperature, humidity, the pitch and slope of the interference fringes, the material thickness, and the exposure dose depending on the recording material, thereby causing wavefront aberrations that are difficult to model accurately owing to the mixing of these factors on the recording material^34–37^. This wavefront aberration is further complicated by other multiple factors, including fabrication errors of the DOEs, such as the quantization error in digital-to-analog conversion or distortion in the fabrication equipment, and misalignment of the optical components^14,16,38,39^, resulting in unpredictable and unknown wavefront aberration, as shown in Fig. 1. The wavefront aberration generated in this way is further accumulated by stacking multiple layers, which significantly degrades the performance of the D^2^NN. Therefore, it is necessary to establish a method of stabilizing the performance by absorbing a range of unknown wavefront aberrations.

As such a method of stabilizing the performance, we propose aberration-adaptive learning, which can reduce the effect of wavefront aberrations on performance by adapting a D^2^NN model for wavefront aberrations. This method prevents performance degradation even when it is difficult to perfectly predict the unknown wavefront aberration due to multiple factors, since it has the novel feature of adaptability to a wide range of wavefront aberrations within a specific range rather than only to certain wavefront aberrations. Aberration-adaptive learning incorporates wavefront aberrations generated from Zernike polynomials and random numbers in each batch of training into the training process. The modulation distribution is trained to adapt to unknown wavefront aberrations by adding the generated wavefront aberrations to the modulation distribution to be trained during the training process. Consequently, the modulation distribution obtained by aberration-adaptive learning enables stable performance of the D^2^NN independent of fabrication errors.

We evaluated the reflective D^2^NN with aberration-adaptive learning consisting of three layers of phase modulation vHOEs through numerical simulations and optical experiments. In the evaluation, a classification task of handwritten digits called MNIST^40^ was used as a benchmark. As a result, we successfully implemented the D^2^NN with vHOEs, and $77.1\%$ classification accuracy was achieved in the simulations and $76\%$ classification accuracy was achieved in the optical experiments. On the other hand, the classification accuracies of D^2^NN without aberration-adaptive learning were $88.7\%$ for simulation and $18\%$ for optical experiments. These results indicate that aberration-adaptive learning stabilized the performance with wavefront aberrations caused by multiple factors, including fabrication errors such as material shrinkage, wavefront aberrations during fabrication processes, distortion in the fabrication equipment, and thermal expansion caused by the fabrication or installation environment. The aberration-adaptive learning significantly contributed to lower system cost by reducing the required design precision and to stable performance in a wide range of applications by improving the robustness to environment-dependent wavefront aberrations.

## Results

### Experimental setup

**Fig. 2:**
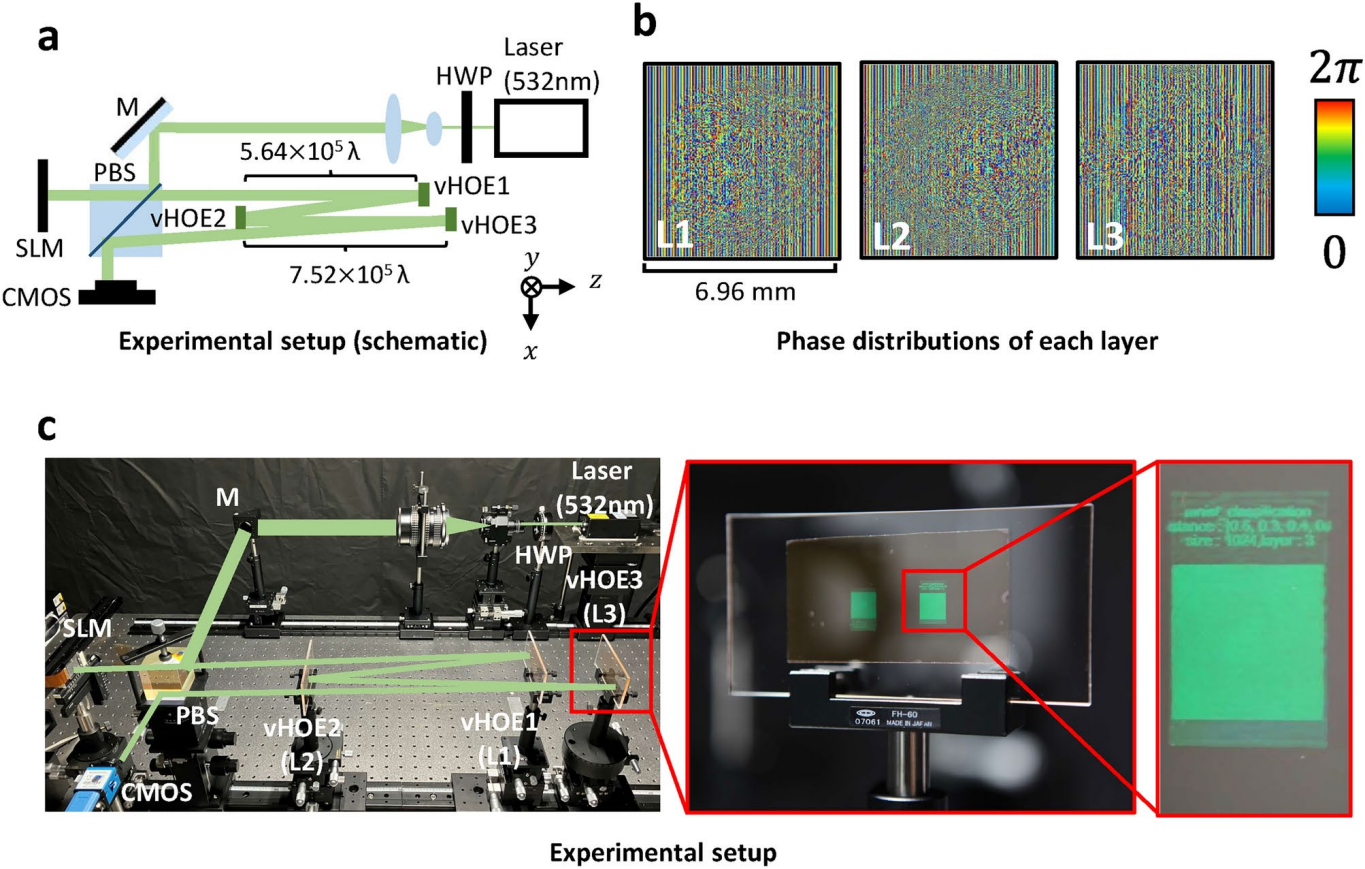
Experimental setup of three-layer D^2^NN with reflective vHOEs. (**a**) Schematic of the experimental setup, where $\lambda$ is $532 \, \text{nm}$. The distance between the SLM and vHOE1 is $1.12 \times 10^6 \lambda$, the distance between vHOE1 and vHOE2 is $5.64 \times 10^5 \lambda$, the distance between vHOE2 and vHOE3 is $7.52 \times 10^5 \lambda$, and the distance between vHOE3 and the complementary metal-oxide-semiconductor (CMOS) is $1.50 \times 10^6 \lambda$. The vHOE2 and the vHOE3 are shifted by $1.32 \times 10^4 \lambda$ from vHOE1 in the x-axis direction. The output wavefront on the CMOS is shifted by $3.52 \times 10^4 \lambda$ from vHOE2 and vHOE3 in the x-axis direction. HWP: half-wave plate, M: mirror, PBS: polarizing beam splitter. (**b**) Phase distribution of each layer obtained by learning. (**c**) Actual optical experimental setup. The following equipment was used. CMOS: The Imaging Source DMK 33UX183, SLM: JVCKENWOOD D-ILA pixel pitch $4.8 \, \upmu \text {m}$, laser: Cobolt $532 \, \text{nm}$ Samba 05. In vHOE3 shown in the photograph, vHOE is inside the red box and the label above vHOE is information about this setup and this layer. The other vHOE outside the red box is a backup sample that does not affect the experiment.

Figure 2 shows the experimental setup of a three-layer D^2^NN with reflective vHOEs. Figure 2a is a schematic of the experimental setup, and Fig 2b shows the phase distributions obtained by *in silico* training. In this setup, only the phase was modulated by the vHOEs. Figure 2c shows the actual optical experimental setup and a photograph of the vHOE3. With the SLM as the input wavefront, the CMOS captures the wavefront reflected from vHOE1, vHOE2, and vHOE3 in this order. In the simulations and optical experiments, the wavelength was $532 \, \text{nm}$. The size of the vHOEs is governed by the pixel pitch and the number of pixels. Here, the pixel pitch is $6.8 \, \upmu \text {m}$, and the number of pixels is $1024 \times 1024 \, \text{pixel}$. Therefore, the size of the vHOEs is $6.96 \, \text{mm}$. The shift in the x-y plane and the distances for each vHOE were determined to prevent overlap between each layer including input and output wavefronts to avoid the influence of direct light. Here, the distance between each layer can be shortened by increasing the diffraction angle because of pixel pitch narrowing.

For training, we used the MNIST^40^ dataset of handwritten numbers expanded to $1024 \times 1024 \, \text{pixel}$ and we trained the function to classify them (details of the computing environment and the training conditions are provided in Sections “Computing environment” and Training conditions). The diffraction efficiency is shown in Fig. S1 in Supplement 1.

### Optical experiments

**Fig. 3:**
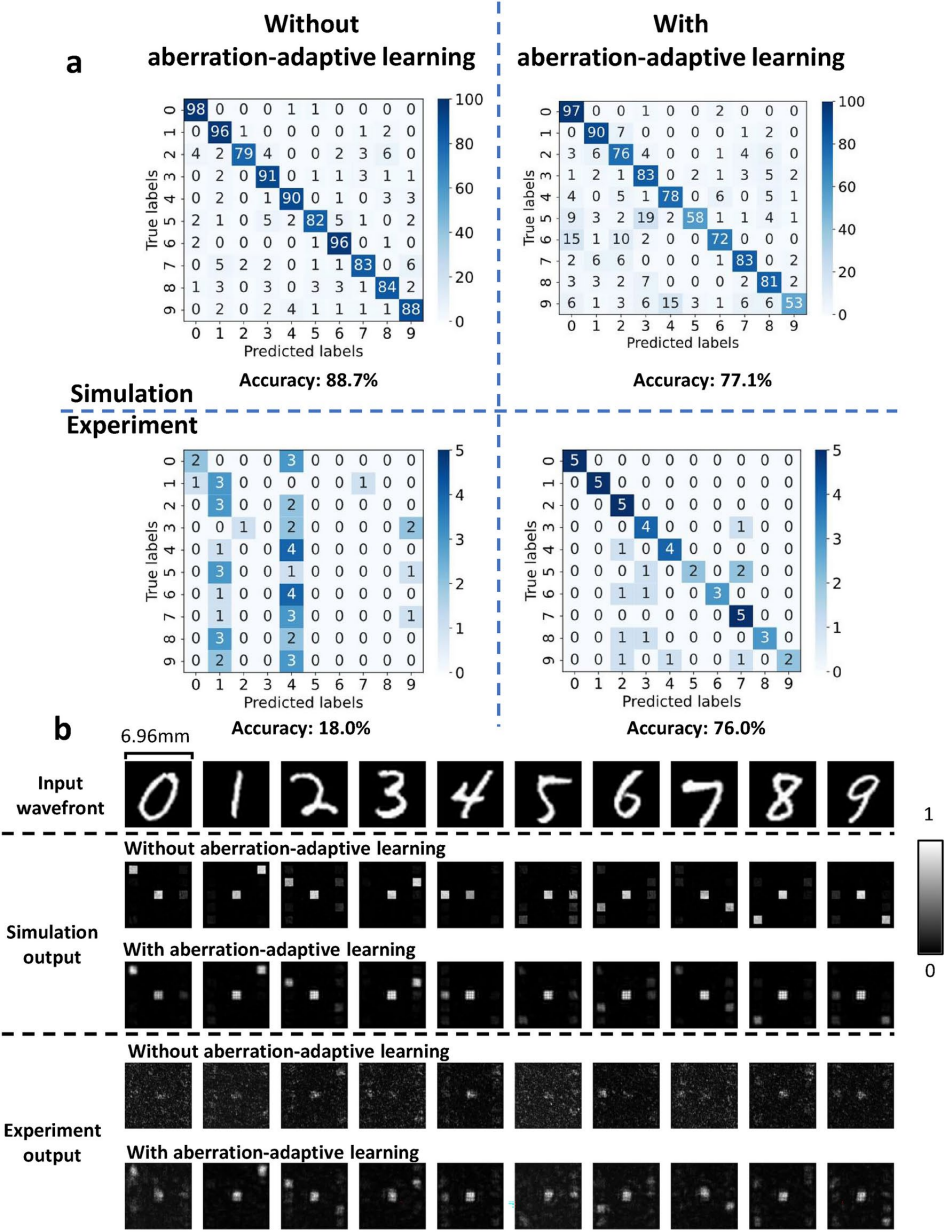
Results of optical experiments and simulations. (**a**) Confusion matrices of the classification results. Each represents the results of simulations and optical experiments without and with aberration-adaptive learning. (**b**) Output wavefront for a given input wavefront for the classification simulation and the optical experiments without and with aberration-adaptive learning. All images are intensity images and padding area of outputs is cropped. Input wavefronts of handwritten digits are classified into the left and right areas corresponding to each number in the output wavefronts. The center area is a calibration marker for preventing misalignment^14,16,38,39^.

Figure 3 shows the results of optical experiments and simulations. The test data were randomly selected without overlap with the training data, the number of simulation test data was 1000, and the number of experiment test data was 50. The accuracy for the simulation of the model without aberration-adaptive learning was $88.7 \%$. Note that this accuracy was the same as the accuracy without the vHOEs shift. Figure 3a shows that the accuracy in the simulation decreases 11.6 percentage points, while the accuracy in the experiment improves 58 percentage points. In addition to improving the accuracy, Fig. 3b shows that the output wavefront in the experiment also becomes closer to that in the simulation, with noise being reduced by aberration-adaptive learning. All the output wavefronts obtained in the optical experiments are shown in Figs. S2 and S3 of the Supplement 1.

**Fig. 4:**
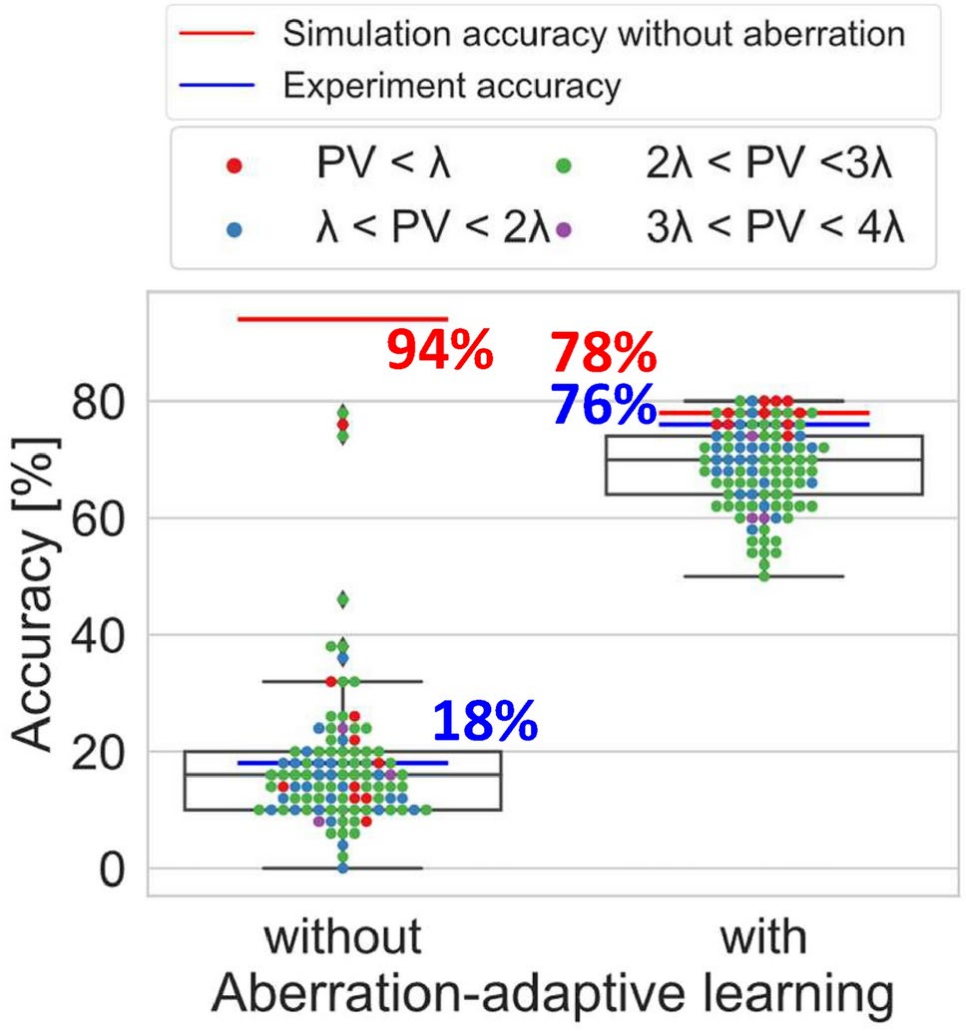
Simulation results of aberration-adaptive learning for the models used in the optical experiment. Each colored point represents the classification accuracies of one out of 100 trials and the strength of the added wavefront aberration, and the box plots represent the results of 100 trials. The red lines represent the accuracies without wavefront aberration, and the blue lines are the experimental accuracies already shown in Fig. 3, shown here for reference. In the box plots, the maximum and the minimum are limited to the interquartile range $\times 1.5$, and values beyond that are considered outliers.

The validity of these results is demonstrated by the comparative simulations without and with aberration-adaptive learning in the case of adding wavefront aberrations, shown in Fig. 4. This simulation was run 100 times with different random wavefront aberrations generated from Zernike polynomials of orders 4 to 6 and a uniform random number from $-0.5$ to 0.5, and the same wavefront aberration for all layers in each run. The peak-to-valley (PV) distances means the difference between the maximum and minimum values of the added wavefront aberration. The test data are the same as those used in the optical experiment. Figure 4 shows that the accuracies of the optical experiments are within the range of the box plots both without and with aberration-adaptive learning. Thus, the results of the optical experiments are considered reasonable since they agree with the simulations to a fair extent. In addition, with aberration-adaptive learning, the accuracy without wavefront aberration is no longer an outlier in the box plot. This is partly due to the reduction in the accuracy without wavefront aberration but is also a result of the upward shift of the box plot area. These results so far confirm the effectiveness of aberration-adaptive learning and the successful implementation of the D^2^NN with vHOEs.

### Off-axis D^2^NN

**Fig. 5:**
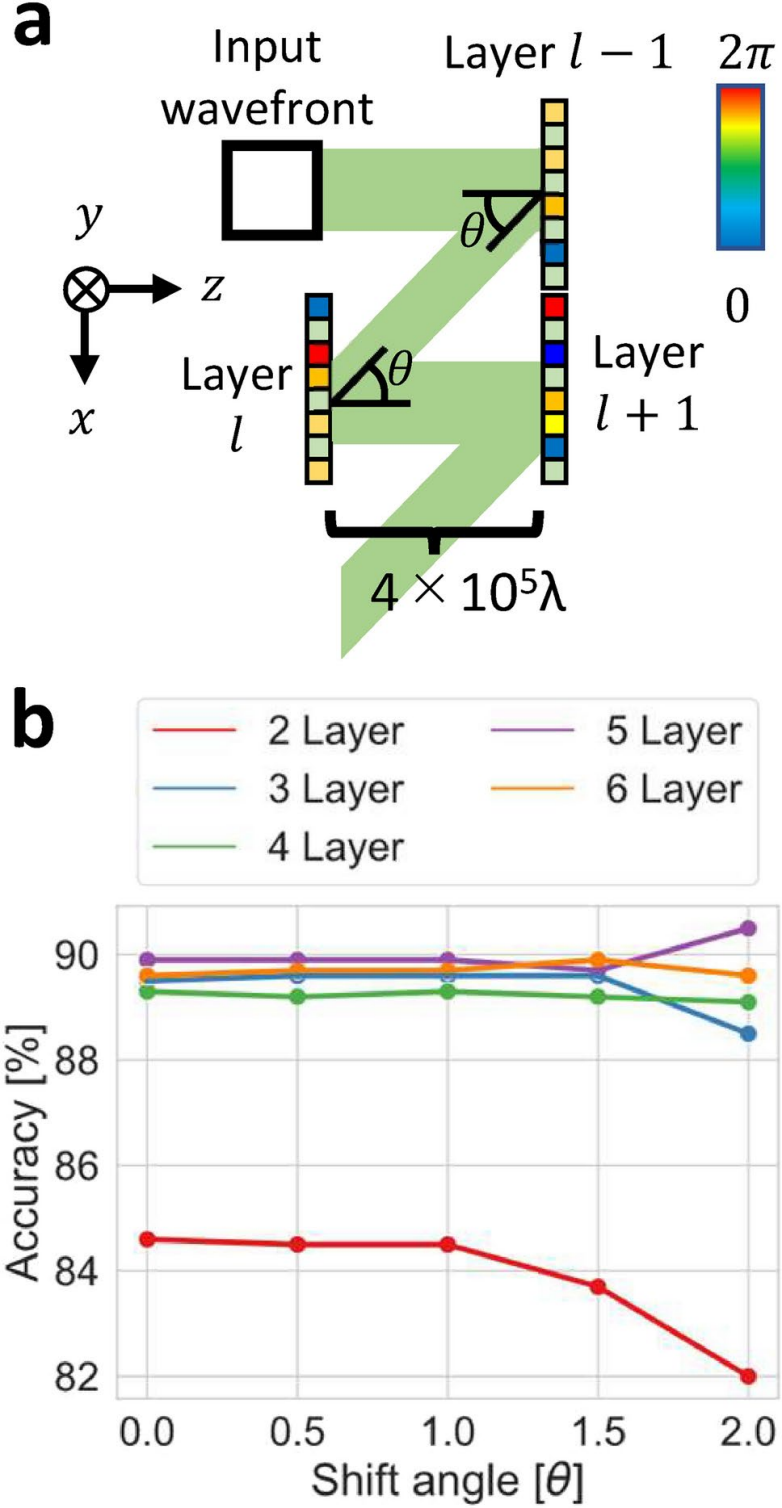
Simulation results of off-axis D^2^NN. (**a**) Simulation model of off-axis D^2^NN. Multiple models were created, trained, and evaluated for each angle and number of layers. (**b**) Graph of accuracies for shift angle $\theta$, where the shift angle $\theta$ corresponds to $\theta$ in (**a**), and each color represents the number of model layers. The accuracy was plotted for $\theta = [0.0^\circ , 0.5^\circ , 1.0^\circ , 1.5^\circ , 2.0^\circ ]$.

In constructing the reflective D^2^NN, we evaluated whether training could be performed without any problems with the shifted layers. Figure 5 shows the results of the evaluation. The model shown in Fig. 5a was trained by shifting the shift angle as $\theta = [0.0^\circ , 0.5^\circ , 1.0^\circ , 1.5^\circ , 2.0 ^\circ ]$, since the maximum diffraction angle is $\sin ^{-1}\left( \frac{\lambda }{2p} \right) =2.24^\circ$ where *p* denotes pixel pitch, and the classification accuracy was evaluated. Here, $\theta = 0.0^\circ$ is equivalent to an inline D^2^NN. From Fig. 5b, we can see that the accuracies are close to those of inline models even when each model layer is shifted, which indicates that the models were trained without any problems. On the other hand, depending on the number of model layers, we can see that the accuracies decrease with increasing shift angle $\theta$. This decrease in accuracy was probably caused by a part of the shifted next layer being outside the maximum diffraction angle. (See the Section “In silico training” for more details).

### Aberration-adaptive learning

**Fig. 6:**
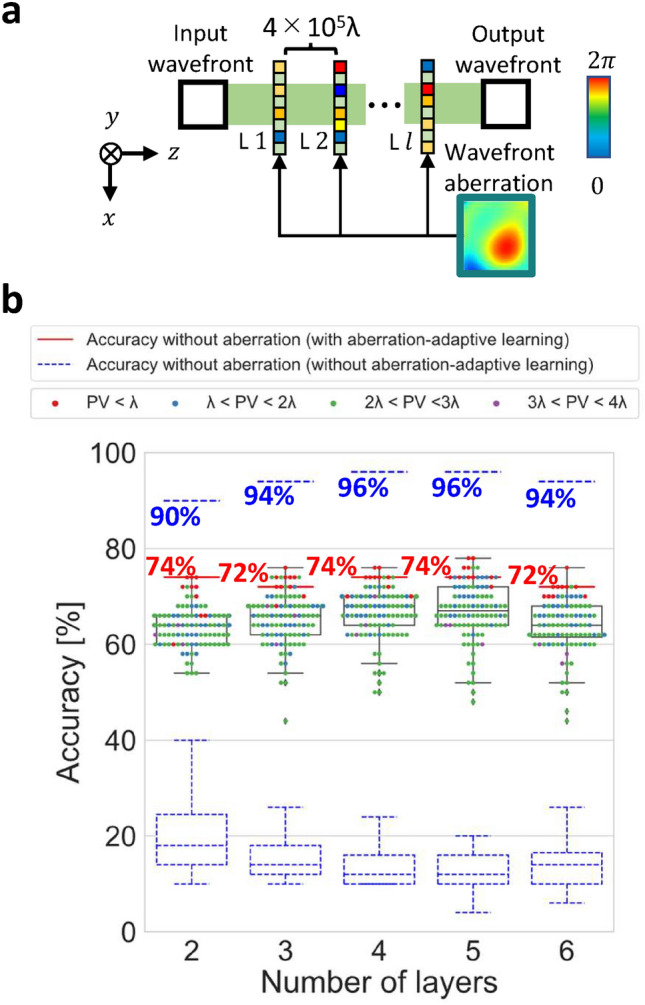
(**a**) Inline D^2^NN model for performance evaluation. The distances between each layer are $4.0 \times 10^5 \lambda$. (**b**) Simulation results of aberration-adaptive learning. Each colored point represents the classification accuracies of one out of 100 trials and the strength of the added wavefront aberration, and the box plots represent the results of 100 trials. The red lines represent the accuracies without wavefront aberration, the blue dashed lines are the accuracies without aberration-adaptive learning or wavefront aberration, and the blue dashed box plots are the results of 100 trials without aberration-adaptive learning, shown here for reference. In the box plots, the maximum and the minimum are limited to the interquartile range $\times 1.5$, and values beyond that are considered outliers.

Figure 6 shows the simulation results of aberration-adaptive learning. These simulation conditions are the same as the simulation shown in Fig. 4. The model as shown in Fig. 6a was retrained using aberration-adaptive learning with a learning rate of 0.1 after an initial training with a learning rate of 0.5 without aberration-adaptive learning. From Fig. 6b, we can see that the accuracies without wavefront aberrations are better in the case without aberration-adaptive learning; however, the overall upward shift of the box plots indicates that the accuracies with wavefront aberrations are better with aberration-adaptive learning. Figure 6b indicates that the gap between the cases with and without wavefront aberration noise is reduced by aberration-adaptive learning. As a result, the accuracy with wavefront aberration improves and becomes closer to the accuracy in the case without wavefront aberration although the maximum accuracy decreases.

## Discussion and conclusion

In this paper, we proposed a D^2^NN implemented by reflective vHOEs with aberration-adaptive learning, and we evaluated it by both simulations and optical experiments. As a result, we have successfully stabilized the performance using aberration-adaptive learning and implemented the D^2^NN with vHOEs. Although the pixel pitch in the optical experiments of this study was $6.8 \, \upmu \text {m}$ owing to the recording conditions, the propagation distance could be significantly shortened by further narrowing the pixel pitch. Fortunately, the pixel pitch of a vHOE can be reduced easily since reduction recording is possible in the recording of vHOEs. In addition, aberration-adaptive learning has significantly reduced the accuracy gap between simulation and experiment of the D^2^NN with vHOEs but it still exists, and an accuracy of $76\%$ is not high compared with simulation without aberration-adaptive learning and other methods^14,15^. Therefore, the accuracy of aberration-adaptive learning also needs to be improved, for example, by increasing the amount of training data through *in silico* training optimization or selecting more appropriate parameters for aberration-adaptive learning. Furthermore, we consider that aberration-adaptive learning is also effective for DOEs other than vHOEs as well, and its effectiveness needs to be evaluated.

Aberration-adaptive learning provides D^2^NNs with high robustness and stability against wavefront distortion, which will accelerate the integration of D^2^NNs into optical systems in various fields, such as adaptive optics for the astronomy and microscopy fields, optical computing, and optical communication devices. In addition, the vHOEs used as DOEs in this work have a wavelength and angle selectivity and multiplexing recordability, as mentioned in the introduction section. This means that D^2^NN with vHOEs can be multiplexed in both wavelength and angle directions to expand optical functionality and information density. These features contribute to the realization of full-color D^2^NN and large-scale parallel computing with wavelength- and angle-multiplexed D^2^NN, thus expanding its functionality in various fields such as optical classifiers, cytometric devices, and mode dividers in optical communications.

## Methods

### In silico training

**Fig. 7:**
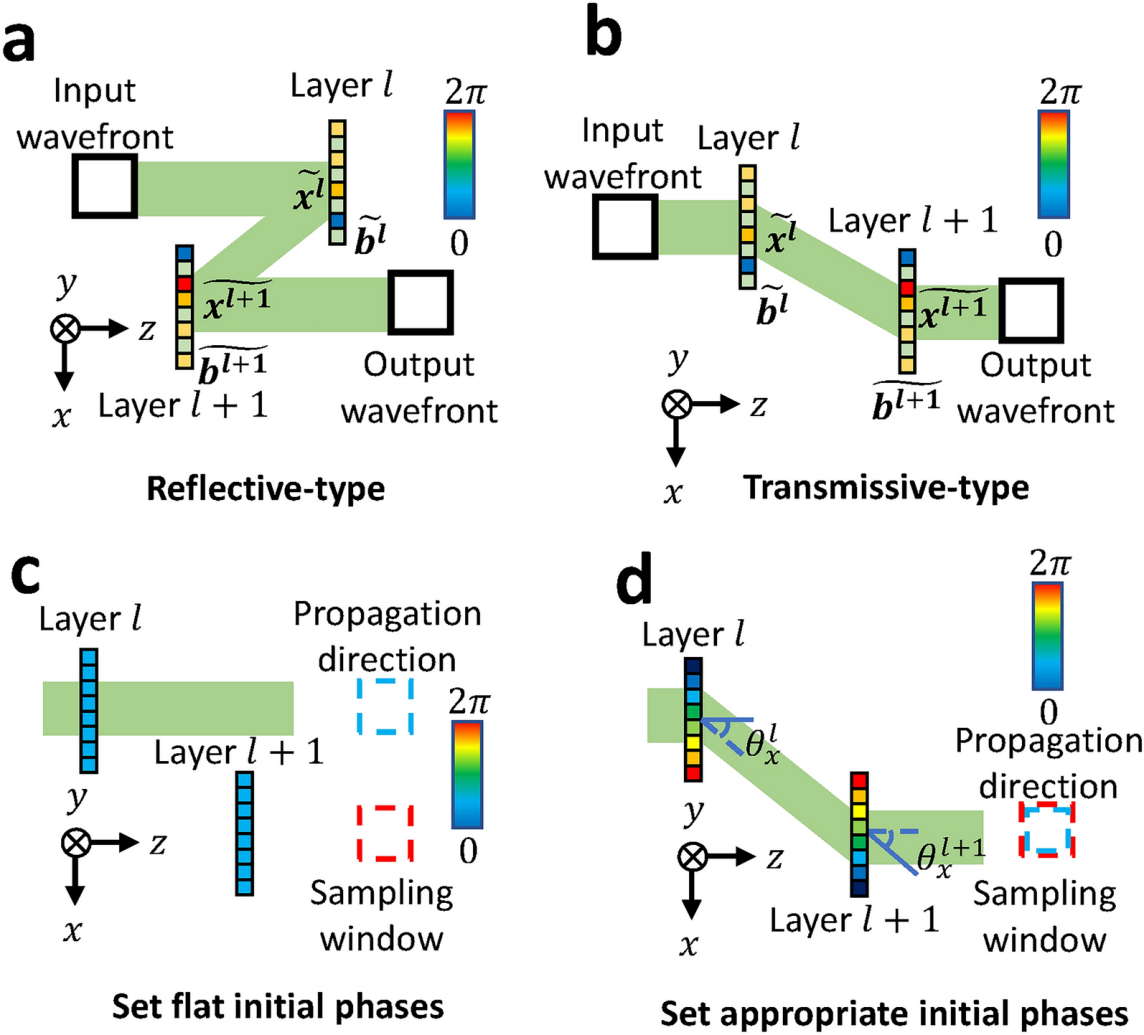
Schematic of off-axis D^2^NN. (**a**) Reflective-type off-axis D^2^NN. (**b**) Transmissive-type off-axis D^2^NN. (**c**) In the case of flat initial phases where all distributions are zero radians, the light propagation direction and the observation window do not match. (**e**) When the appropriate initial phases are set, the light propagation direction and the observation window match.

A D^2^NN with vHOEs requires *in silico* training, in which the propagation and diffraction are calculated on a computer and the phase modulation is updated, because vHOEs are static elements. Generally, the angular spectrum method^41^, in which the wavefront is calculated when a given wavefront propagates by an arbitrary distance, and other methods are used to calculate the propagation in D^2^NNs. However, in this study, we used the shifted angular spectrum method^42^ to use reflective vHOEs and to avoid the effect of direct light that is reflected or transmitted without diffraction by the vHOEs. This method is a propagation calculation based on the angular spectrum method and can calculate the wavefront in any x-y plane in addition to any distance as follows:

1 $$\begin{aligned} \hat{g}= & \mathcal {F}^{-1} \left[ \mathcal {F} \left[ g \right] \exp \left[ i 2 \pi \left( x_0 u + y_0 v + z_0 w(u,v) \right) \right] \right] , \end{aligned}$$

2 $$\begin{aligned} w(u,v)= & \left( \lambda ^{-2} - u^2 - v^2 \right) ^{1/2}, \end{aligned}$$

where $g$ and $\hat{g}$ represent the wavefront before propagation and at the destination, respectively. $\mathcal {F}$ represents the Fourier transform. *i* denotes an imaginary unit. *u* and *v* are Fourier frequencies. $x_0$, $y_0$, and $z_0$ are the coordinates of the destination wavefront, where $z_0$ denotes the propagation distance and $x_0$ and $y_0$ are the coordinates of the wavefront in the destination plane at $z_0$. $\lambda$ denotes the wavelength. When *w*(*u*, *v*) becomes imaginary, the wavefronts are exponentially decayed after $z>0$. These waves are called evanescent, and they propagate over a distance of only about a wavelength. When $x_0=0$ and $y_0=0$, the shifted angular spectrum method is the same as the angular spectrum method. The shifted angular spectrum method can be used to design off-axis reflective D^2^NNs, as shown in Fig. 7a, and off-axis transparent D^2^NNs, as shown in Fig. 7b. A folded structure^43^ is also possible by repeating the structure shown in Fig. 7a.

Here, the wavefront $\widetilde{x^{l+1}}$ of the $l+1$th layer in the off-axis D^2^NN is represented as

3 $$\begin{aligned} \widetilde{x^{l+1}} = \mathcal {F}^{-1} \left[ \mathcal {F} \left[ \widetilde{x^l} \widetilde{b^l}\right] \exp \left[ i 2 \pi \left( x_0 u + y_0 v + z_0 w \right) \right] \right] , \end{aligned}$$

where $\widetilde{x^l}$ is the wavefront of the *l*th layer and $\widetilde{b^l}$ is the phase modulation of the *l*th layer; $\widetilde{b^l} = e^{i\psi }$, as $\psi$ is the amount of phase modulation.

Note that the shifted angular spectrum method is only used to shift the sampling window, not to propagate the wavefront in the desired direction. Therefore, the propagation direction of the wavefront is outside the sampling window in the early stages of learning, as shown in Fig. 7c, and the learning falls into an unintended local optimal solution. The off-axis D^2^NN cannot train without resolving this issue. We configure appropriate initial phases to resolve this issue. In this way, wavefronts are propagated to the next layer correctly, as shown in Fig. 7d, even in the early stages of training. Since the appropriate initial phases should be set so that the wavefront reaches the next layer, they are expressed using angles $\theta ^l _x, \theta ^l _y$ as follows:

4 $$\begin{aligned} \theta (x,y) = \theta _0 + kx \sin \left( \theta ^l _x \right) + ky \sin \left( \theta ^l _y \right) . \end{aligned}$$

$\theta ^l _x, \theta ^l _y$ are the angles of the differences between the wavefront propagation direction before and after passing through the layer along the x- and y-axes, respectively. $\theta _0$ is a constant below $2 \pi$. *k* denotes the wave number, and $k = 2 \pi / \lambda$. $\theta ^l _x$ and $\theta ^l _y$ are obtained from the distance and shift of each layer, respectively. In this study, the size of the vHOEs in each layer is unified. If the size of the vHOEs in each layer is different, focusing or divergence phases are given in addition to the initial phases.

**Fig. 8:**
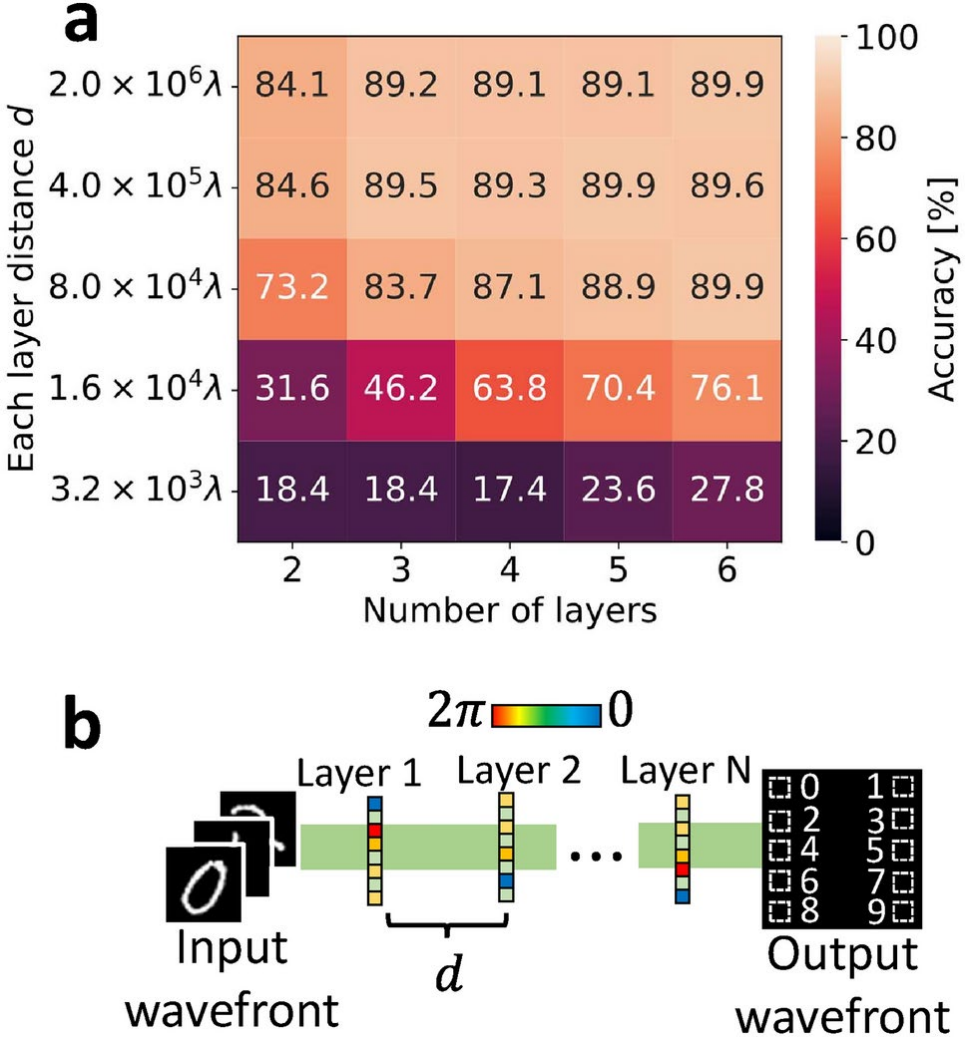
(**a**) Performance at different distances and number of layers. (**b**) Model for performance evaluation.

For off-axis D^2^NN, the relationship between the amount of shift and the propagation distance should be noted. In the case of general D^2^NNs, the distance constraint on whether the diffraction spot completely contains the next layer is represented as

5 $$\begin{aligned} d \ge W \cdot p \times \sqrt{\frac{4p^2}{\lambda ^2} - 1}, \end{aligned}$$

where *d* represents the distance between layers, *W* represents the number of pixels in the width direction, and *p* represents the pixel pitch. Figure 8a shows the performance of the simple model shown in Fig. 8b at different distances and number of layers. The shift amounts were zero, i.e., the inline model was trained. The test data were randomly selected without overlap with the training data, and the number of data was 1000. Equation (5) is satisfied for distances of $4 \times 10^5 \lambda$ and greater, since the number of pixels in the width direction is $1024 \, \text{pixel}$, the pixel pitch is $6.8 \, \upmu \text {m}$, and the wavelength is $532 \, \text{nm}$. Figure 8a shows that the performance is degraded when the constraint of Eq. (5) is not satisfied. Since Eq. (5) is a constraint on whether the diffraction spot completely contains the next layer, it is necessary to consider the shift amount $x_0$ in the off-axis D^2^NN as follows:

6 $$\begin{aligned} d \ge \left( W \cdot p + x_0 \right) \times \sqrt{\frac{4p^2}{\lambda ^2} - 1}. \end{aligned}$$

Performance may degrade if this equation is not satisfied. Here, Eq. (6) shows that $x_0 < 1.4 \times 10^{-3}$ and $\theta <0.37$ for the model used as desctibed in Sect. 2.3. From these conditions and Fig. 6, it is confirmed that the failure to satisfy Eq. (6) causes performance degradation, but that performance is not immediately degraded when Eq. (6) is not satisfied.

### Computing environment

The computing environment for learning was as follows: the CPU was Intel$^\text {\text{\textregistered} }$ Xeon$^\text {\text{\textregistered} }$ CPU E5-2620 v4 (2.10 GHz), the GPU was NVIDIA$^\text {\text{\textregistered} }$ Quadro$^\text {\text{\textregistered} }$ GV100, the memory was 256 GB, the OS was Windows 10 Pro, and the language was python 3.8. TensorFlow 2.4.0 and Keras 2.4.3 were adopted as the library for machine learning. CUDA 11.3 and cuDNN 8.1.4 is adopted as the GPU execution environment.

### Training conditions

We used handwritten digit images, called MNIST, and resized them to $1024 \times 1024 \, \text{pixel}$. The teaching data for the output wavefront were created with $512 \times 512 \, \text{pixel}$ and zero padded to $1024 \times 1024 \, \text{pixel}$. The amount of data used was 5000 for training data and 1000 for evaluation data, with both types of selected at random. The batch size was 50, the loss function was the mean squared error, and the optimization function was Adam^44^ with hyperparameters $\beta _{1} = 0.9$, and $\beta _{2} = 0.999$. A learning rate was set 0.1 in Section “Optical experiments” and 0.5 in Section “Off-axis D^2^NN”. Early stopping was used, and training was terminated when convergence was achieved. The simulation results in Section “Optical experiments”, “Off-axis D^2^NN”, “Aberration-adaptive learning”, which were also trained on FASHION MNIST^45^ under the same conditions, are shown in Figs. S4–S6 in the supplement 1.

### Aberration adaptive learning

**Fig. 9:**
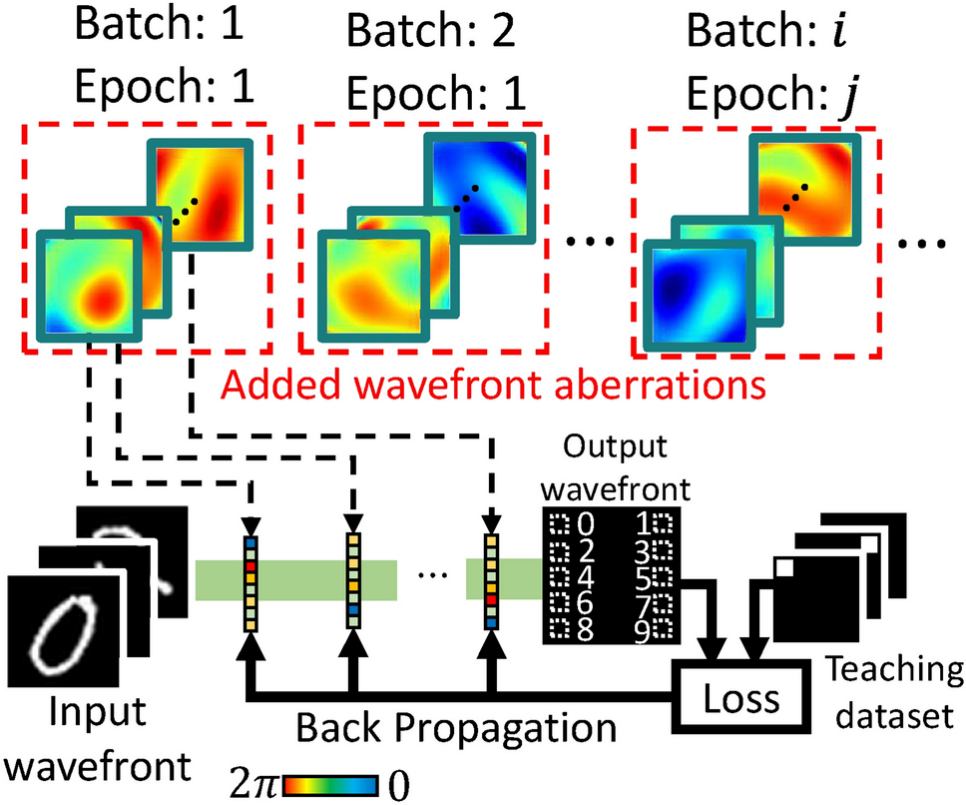
Aberration-adaptive learning concept. Different wavefront aberrations are generated and added to each layer in each batch and epoch.

Fabrication errors are one of the main causes of performance degradation of a D^2^NN, not just vHOEs, as they cause wavefront aberration and distortion of the phase modulation, resulting in performance degradation. It is almost certain that the performance is significantly affected by additional wavefront aberrations, as already shown in Fig. 6b. Nevertheless, as described in the Section “Introduction”, the aberrations are difficult to predict and cannot be completely eliminated. Therefore, it is necessary to make the D^2^NN with vHOEs tolerant to unknown wavefront aberrations. Thus, we incorporated a wavefront aberration generated using Zernike polynomials and random numbers that follow a probability distribution into the learning model to adapt the D^2^NN to the wavefront aberrations due to fabrication errors, as shown in Fig. 9. Zernike polynomials are orthogonal polynomials defined on the unit circle, where a lower-order term corresponds to a Seidel aberration. Arbitrary wavefronts can be represented by a linear combination of Zernike polynomials. In this study, incorporated wavefront aberrations were used to generate different wavefront aberrations for each batch and epoch using the Zernike polynomials of orders 4 to 6 corresponding to oblique astigmatism, defocus, and vertical astigmatism. The coefficients of the Zernike polynomials were randomly generated, and a different wavefront aberration was added to the phase modulation for each batch. More detailed diagrams related to aberration-adaptive learning are shown in Fig. S7 in the supplement 1.

The phase modulation of the *l*th vHOE when incorporating wavefront aberration is represented as $\widetilde{x^l} \widetilde{b^l} e^{i \varphi } = \widetilde{x^l} e^{i ( \psi + \varphi )}$, where $e^{i\varphi }$ is the incorporated wavefront aberration. The strength and range of wavefront aberrations that can be adapted are determined by the type of probability distribution and the parameters in the aberration-adaptive learning; training does not progress well if the wavefront aberration to be considered is too strong or too wide. In our configuration, the random numbers were generated from a distribution combining two Gaussian distributions with a variance of $0.05 \times 2\pi$, and a mean of $+0.3 \times 2\pi$ and $-0.3 \times 2\pi$, so that the mean of the PV distances was between $2 \lambda$ and $3 \lambda$. The parameters and distributions were determined by measuring the wavefront aberration of an actual vHOE or the vHOE fabrication system with a wavefront sensor so that a close approximation of the wavefront aberration could be generated. Generated random number and PV value distributions are shown in Fig. S8 in the supplement 1. For a discussion of parameter selection, see Section 4.2 and Fig. S9 in the supplement 1.

Aberration-adaptive learning may not proceed depending on the D^2^NN model structure, learning task, and strength or range of wavefront aberration. Two-step learning may be effective in such cases. In two-step learning, task training and adaption to the wavefront aberration are performed separately. After first training a D^2^NN model without wavefront aberrations, the model is retrained with aberration-adaptive learning on the same data set. The model constructed in this study is adapted to wavefront aberrations by two-step learning.

### Volume holographic optical element

**Fig. 10:**
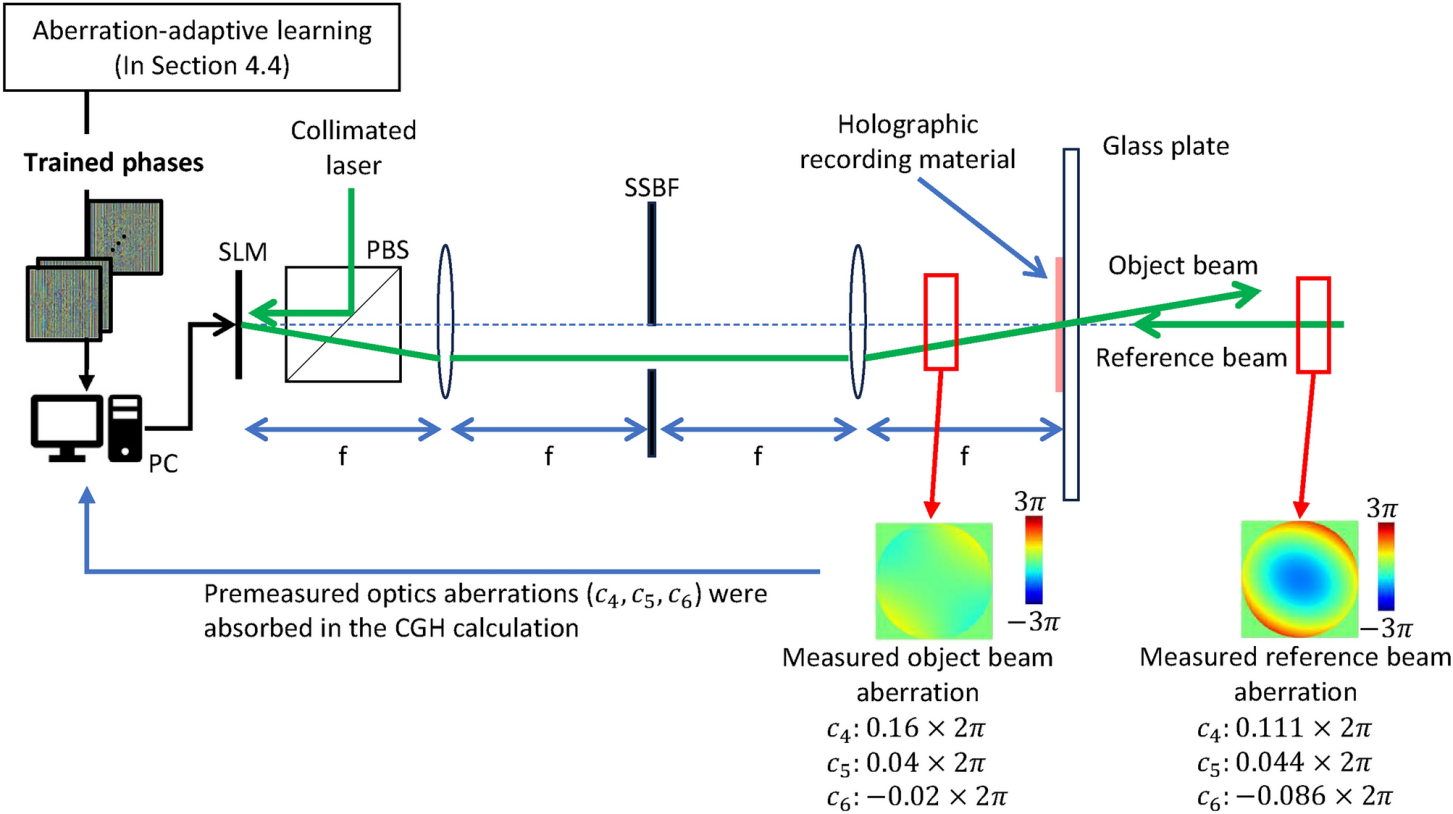
Schematic of optics of wavefront printer. c4, c5, and c6 correspond to the following Zernike polynomial coefficients: c4 oblique astigmatism, c5 defocus, and c6 vertical astigmatism. Each coefficient is the value when the vHOEs are fabricated for the experiments described in Sections “Experimental setup”, “Optical experiments”. *SSBF* single-sideband filter.

The vHOEs were fabricated by a wavefront printer that directly exposed the recording material to a wavefront optically reproduced using a computer-generated hologram (CGH)^46–50^. A schematic of the optics of the wavefront printer is shown in Fig. 10. A phase distribution was encoded to the amplitude CGH by calculating its cosine and then displayed on an amplitude-type SLM. A collimated laser (recording wavelength: 532 nm) was input to the SLM, and interference fringes were formed with the object beam and the collimated reference beam. The object beam was filtered and reimaged by a 4f system and a single-sideband filter to remove the undiffracted beam, conjugate beam, and higher-order diffracted beam. The formed interference fringes were recorded as a reflection hologram on hologram-recording material (Covestro Bayfol HX-200 photopolymer material). The thickness of the photopolymer layer of this material is $16 \pm 2 \, \upmu \text {m}$. Since this thickness considerably affects the diffraction efficiency and the angular and wavelength selectivity of the vHOE, it will be necessary in the future to design it appropriately in accordance with the light utilization efficiency required for the overall optical system, the angular error allowed for alignment, and the wavelength range of the regenerative light source. To prevent the dominant wavefront aberration factors due to the optical system, before recording, the wavefront aberration at the carrier frequency of the object beam and reference beam was measured using a Shack–Hartmann wavefront sensor (Thorlabs WFS20-5C), and measured wavefront aberrations $\Phi ' (\varvec{c})$ were subtracted from the phase distribution $\psi$ to be recorded, to obtain a phase distribution of $\psi - \Phi ' (\varvec{c})$. $\varvec{c}$ represents Zernike polynomial coefficients measured by the wavefront sensor. The wavefront aberration $\Phi (\varvec{c})$ was added when recording, and the phase distribution actually recorded is represented as $\psi - \Phi ' (\varvec{c}) + \Phi (\varvec{c}) = \psi + \left( \Phi (\varvec{c}) - \Phi ' (\varvec{c})\right)$. In this study, the wavefront aberrations to be subtracted were generated from the terms corresponding to oblique astigmatism, defocus, and vertical astigmatism of the Zernike polynomials. Therefore, $\varvec{c} = [c_4, c_5, c_6]$, $c_4$ represents oblique astigmatism, $c_5$ represents defocus, and $c_6$ represents vertical astigmatism. Note that it was not possible to remove the wavefront aberration completely, as described in Section “Introduction”, since the wavefront distortions also occur owing to measurement errors of wavefront sensors and shrinkage/expansion of the recording material.

## Supplementary Information

Supplementary Information.

## Declarations

### Data availability

All the data needed to evaluate the conclusions of this work are present in the main text and the Supplementary Materials. Additional data are available from the corresponding author on reasonable request. The datasets used during the current study are only MNIST and fashion-MNIST, which are general datasets. These datasets can be downloaded through TensorFlow.
