# Supplementary material for: Wavefront-aberration-tolerant diffractive deep neural networks using volume holographic optical elements
Source: Sci Rep. 2025 Jan 7;15:1104. doi: 10.1038/s41598-024-82791-z (PMC11707286; doi:10.1038/s41598-024-82791-z)
Supplement: Supplementary file 1 — Supplementary Information. [file 41598_2024_82791_MOESM1_ESM.pdf]

Supplementary information for Wavefront-aberration-tolerant diffractive deep neural networks using volume holographic optical elements

Ikuo Hoshi[1*], Koki Wakunami[1], Yasuyuki Ichihashi[1] and Ryutaro Oi[1]

[1*]Applied Electromagnetic Research Center, National Institute of Information and Communications Technology, Nukui-Kitamachi, Koganei, 184-8795, Tokyo, Japan.

*Corresponding author(s). E-mail(s): hoshi@nict.go.jp; Contributing authors: k.wakunami@nict.go.jp; y-ichihashi@nict.go.jp; oi.ryutaro@nict.go.jp;

This pdf file includes:

Figure S1 – S9.
Algorithm S1 – S3.
Source code S1.

1 diffraction efficiency

Figure S1 (a), (b), and (c) show the transmission spectrum of each layer used for obtaining diffraction efficiencies. The diffraction efficiencies were obtained by calculating $\frac{S_{\mathrm{Base}}-S_{\mathrm{Peak}}}{S_{\mathrm{Base}}}$ from S_{Base} and S_{Peak} in these graphs [1]. For off-axis D^2NN with vHOEs shown in Figs. 2 and 3 in the main text, the diffraction efficiencies were 90.1% for the first layer, 90.0% for the second layer, and 88.8% for the third layer. Thus, the overall diffraction efficiency of the three layers was 72.0%. This diffraction efficiency and transparency should be improved by further optimization of the parameters of recording and after processing or by improving the performance of the hologram-recording material.

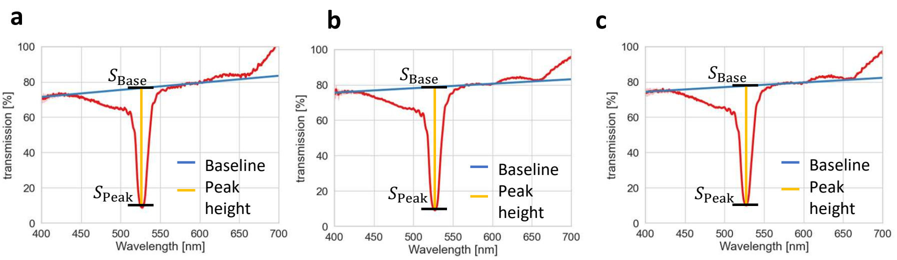

Fig. S1 Transmission spectrum of each layer: (a) first layer, (b) second layer, and (c) third layer.

2 experiment results

Figures S2 and S3 show all the output wavefronts in the optical experiment shown in Fig. 3 without and with aberration-adaptive learning, respectively. A red frame means the correct answer, and numbers below the output wavefront are prediction results.

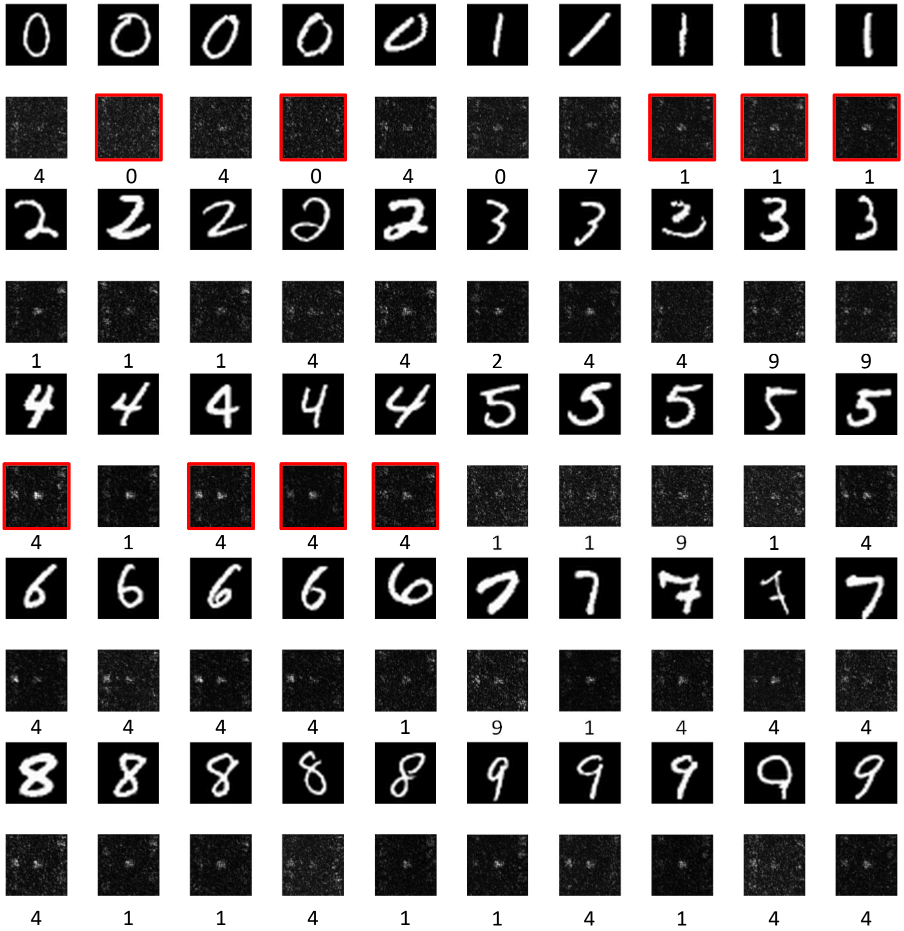

Fig. S2 All output wavefronts in the optical experiment without aberration-adaptive learning.

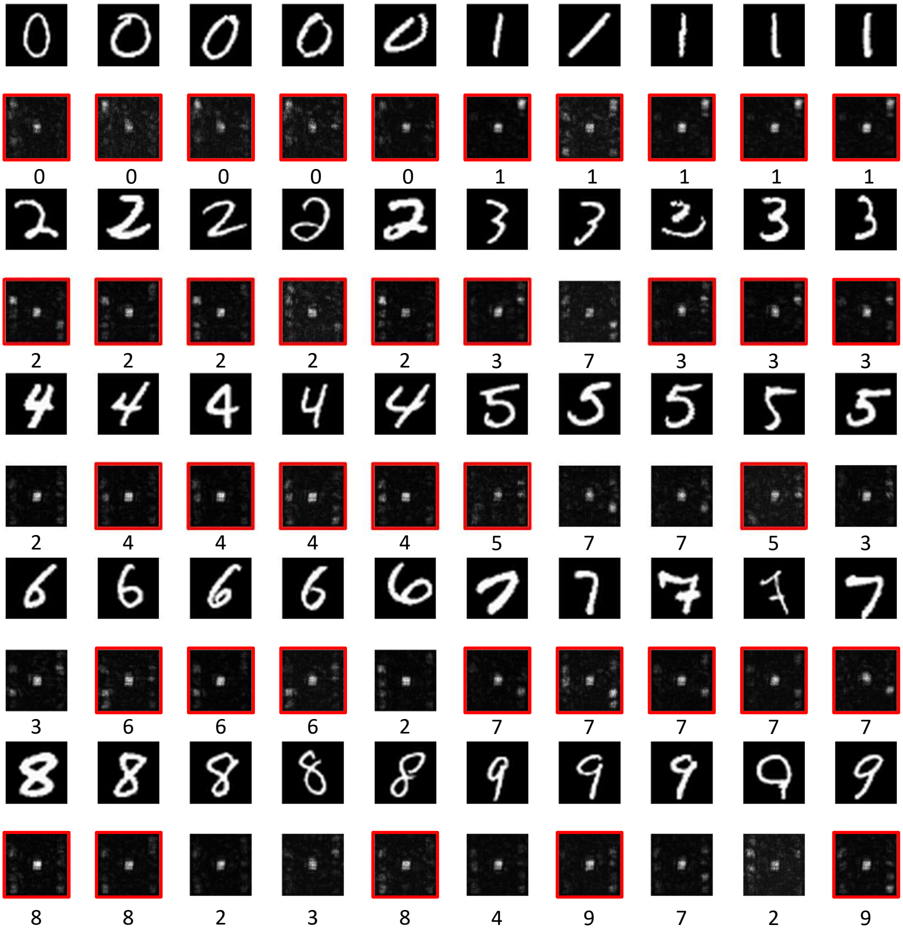

Fig. S3 All output wavefronts in the optical experiment with aberration-adaptive learning.

3 Additional simulations

Figures S4 – S6 show the additional simulations in the case of using fashion mnist as a dataset. Figure S4 corresponds to Section 2.2 in the main text, Figure S5 corresponds to Section 2.3 in the main text, and Figure S6 corresponds to Section 2.4 in the main text, with the same conditions except for the dataset. The training data were resized to 896×896 pixel and then zero-padded to 1024×1024 pixel.

Although the accuracy is degraded in certain classes in Fig. S4, the results are generally similar to those of the mnist case in all simulations. Regarding the performance degradation of certain classes, Figure S4 shows that other classes are classified correctly with aberration-adaptive learning and that the accuracies of classes 2, 5, and 6 are lower than for other classes even without aberration-adaptive learning. In particular, classes 2 and 5 are less confident in their answers because the output wavefronts have less light in the area of the correct answer than the others. This indicates that the classification of these classes is not easy even with the D^2NN without aberration-adaptive learning. Therefore, we consider that our D^2NN requires base performance improvement by increasing the amount of training data or the number of pixels as neurons.

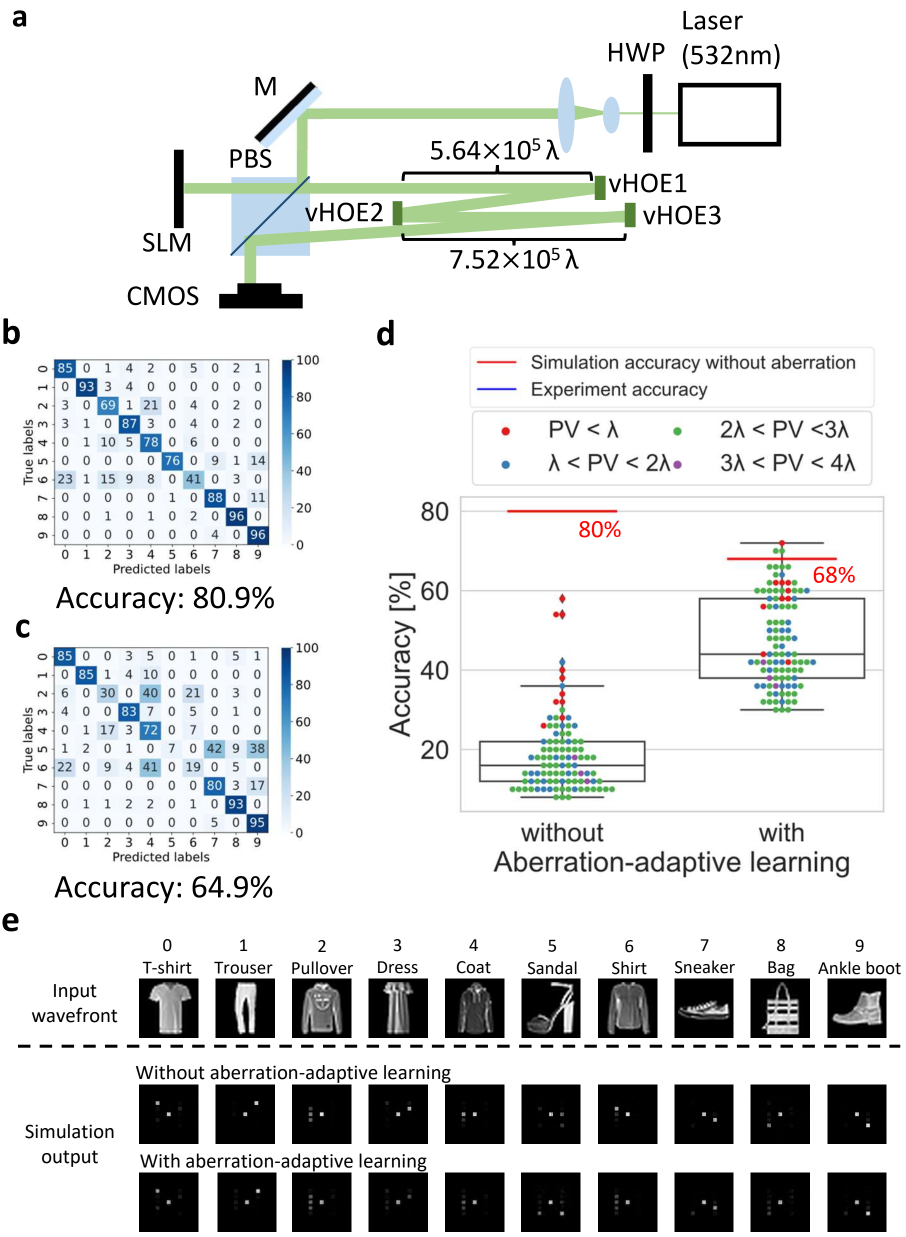

Fig. S4 The simulation result of Section 2.2 in the main text when using fashion mnist as dataset. (a) The simulation model similar to that shown in Fig. 2 in the main text. (b) The confusion matrix of the classification result without aberration-adaptive learning. (c) The confusion matrix of the classification result with aberration-adaptive learning. (d) Simulation results for the aberration-adaptive learning evaluation. Each colored point represents the classification accuracies of one out of 100 trials and the strength of the added wavefront aberration, and the box plots represent the results of 100 trials. The red lines represent the accuracies without wavefront aberration. In the box plots, the maximum and the minimum are limited to the interquartile range $\times 1.5$, and values beyond that are considered outliers. (e) Output wavefront for a given input wavefront for the classification simulation without and with aberration-adaptive learning.

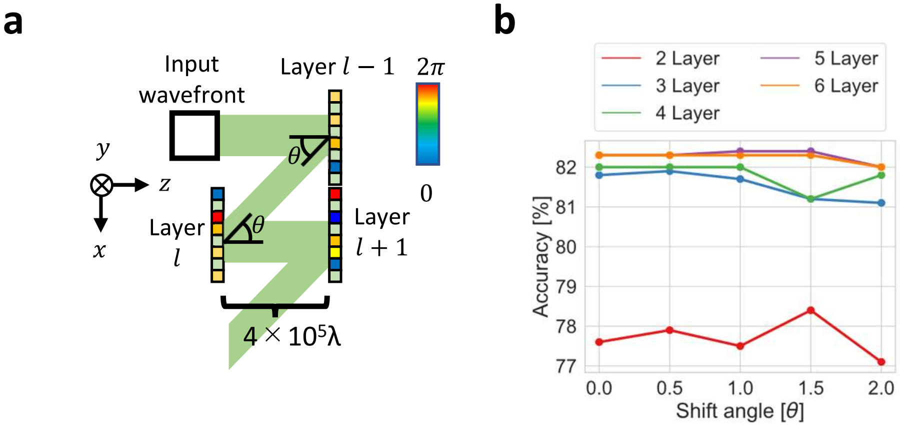

Fig. S5 The simulation result of Section 2.3 in the main text when using fashion mnist as dataset. (a) Simulation model of off-axis D^2NN. Multiple models were created, trained, and evaluated for each angle and number of layers. (b) Graph of accuracies for shift angle θ, where the shift angle θ corresponds to θ in (a), and each color represents the number of model layers. The accuracy was plotted for $\theta = [0.0°, 0.5°, 1.0°, 1.5°, 2.0°]$.

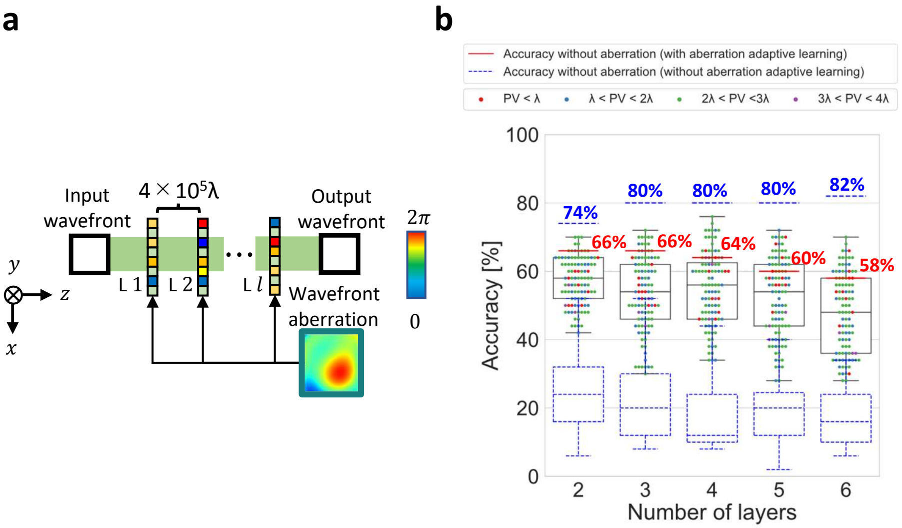

Fig. S6 The simulation result of Section 2.4 in the main text when using fashion mnist as dataset. (a) Inline D^2NN model for performance evaluation. The distances between each layer are $4.0 \times 10^5\lambda$. (b) Simulation results of aberration-adaptive learning. The red lines represent the accuracies without wavefront aberration, each colored point represents the classification accuracy of one out of 100 trials and the strength of the added wavefront aberration, and the box plots represent the results of 100 trials. The blue dashed lines and the box plots are results without aberration-adaptive learning.

4 Aberration-adaptive learning

4.1 Model architecture

Figure S7 shows two-step learning. In the case of single-step learning, the initial phases are set instead of the phases obtained by learning in the first step. In aberration-adaptive learning, the wavefront aberration added layer is inserted after the diffraction layer. By doing this, the variable of the modulation amount to be learned can be adapted to wavefront aberrations since the learning process proceeds with the wavefront aberration overlapping the modulation amount. A sample source code of the model definition is shown in source code S1 and the algorithm for each layer in Fig. S7 is shown in Algorithm S1 – S3.

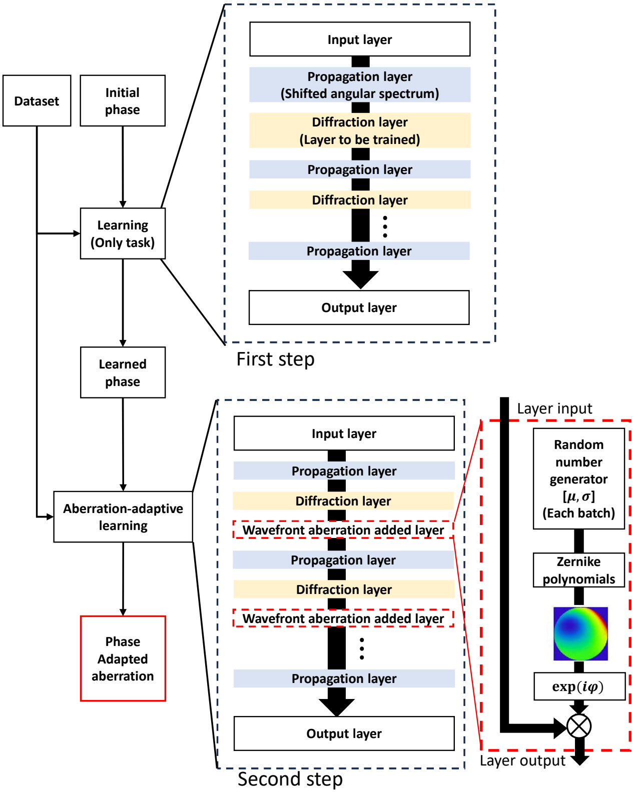

Fig. S7 Network structure of aberration-adaptive learning.

source code S1 sample code of model definition

```
from keras.models import Model
from keras.layers import Input
from customlayer.ShiftedAngularSpectrum \
import ShiftedAngularSpectrum
from customlayer.Diffractive import Diffractive
from customlayer.AddWavefrontAberration \
import AddWavefrontAberration
from keras.layers import ZeroPadding2D, Cropping2D

def CreateModel(input_shape, wave_lambda, distance_list,
                x_offset_list, y_offset_list, trainable_list,
                noise_mean, noise_std, pitch, optimizer, loss):

  layer_num = len(distance_list) - 1
  inputs = Input(shape = (input_shape[1],
                          input_shape[2],
                          input_shape[3]),
                 dtype = 'complex64')

  x = ZeroPadding2D(padding = (input_shape[2] // 2,
                               input_shape[3] // 2),
                    data_format = "channels_first")(inputs)

  for i in range (layer_num):
      distance = distance_list[i]
      x_offset = x_offset_list[i]
      y_offset = y_offset_list[i]
      trainable = trainable_list[i]

      if(i != 0):
          x = ZeroPadding2D(padding = (input_shape[2] // 2,
                                       input_shape[3] // 2),
                            data_format = "channels_first")(x)
      x = ShiftedAngularSpectrum(wave_lambda,
                                 distance,
                                 pitch,
                                 x_offset = x_offset,
                                 y_offset = y_offset)(x)
      x = Cropping2D(cropping = (input_shape[2] // 2,
                                 input_shape[3] // 2),
                     data_format = "channels_first")(x)
      x = Diffractive(trainable = trainable)(x)
      x = AddWavefrontAberration(noise_mean = noise_mean,
                                 noise_std = noise_std)(x)
```

```
distance = distance_list[-1]
x_offset = x_offset_list[-1]
y_offset = y_offset_list[-1]

x = ZeroPadding2D(padding = (input_shape[2] // 2,
                             input_shape[3] // 2),
                  data_format = "channels_first")(x)
x = ShiftedAngularSpectrum(wave_lambda,
                           distance,
                           pitch,
                           x_offset = x_offset,
                           y_offset = y_offset)(x)
x = Cropping2D(cropping = (input_shape[2] // 2,
                           input_shape[3] // 2),
               data_format = "channels_first")(x)

model = Model(inputs = inputs, outputs = x)
model.compile(optimizer=optimizer,
              loss=loss)
return model
```

Algorithm S1 Propagation layer (Shifted angular spectrum [2])

Input: $\widetilde{x}_l, x_0, y_0, z_0, width, height, p, \lambda, \Delta u, \Delta v$
// *width* and *height* are values after padding.
Output: $\widetilde{x}_{l+1}$
Initialization:
$u = -\frac{1}{2p}, -\frac{1}{2p} + \Delta u, \ldots, \frac{1}{2p}$
$v = -\frac{1}{2p}, -\frac{1}{2p} + \Delta v, \ldots, \frac{1}{2p}$
$u^{\pm}_{limit} = \left[\left(x_0 \pm \frac{1}{2\Delta u}\right)^{-2} z_0^2 + 1\right]^{-\frac{1}{2}} \lambda^{-1}$
$v^{\pm}_{limit} = \left[\left(y_0 \pm \frac{1}{2\Delta v}\right)^{-2} z_0^2 + 1\right]^{-\frac{1}{2}} \lambda^{-1}$
for u, v do
$w = \left(\lambda^{-2} - u^2 - v^2\right)^{1/2}$
//The case when the w becomes imaginary is not cared about because
//the w does not become imaginary in the parameters of this study.
$H(u,v) = \exp\left[i2\pi\left(x_0 u + y_0 v + z_0 w\right)\right]$
if $\frac{width}{2}p < x_0$
for u **do if** not $(u^{-}_{limit} \leq u \leq u^{+}_{limit})$ then $H(u,v) = 0$
else if $-\frac{width}{2}p \leq x_0 \leq \frac{width}{2}p$
for u **do if** not $(-u^{-}_{limit} \leq u \leq u^{+}_{limit})$ then $H(u,v) = 0$
else if $x_0 < -\frac{width}{2}p$
for u **do if** not $(-u^{-}_{limit} \leq u \leq -u^{+}_{limit})$ then $H(u,v) = 0$
if $\frac{height}{2}p < y_0$
for v **do if** not $(v^{-}_{limit} \leq v \leq v^{+}_{limit})$ then $H(u,v) = 0$
else if $-\frac{height}{2}p \leq y_0 \leq \frac{height}{2}p$
for v **do if** not $(-v^{-}_{limit} \leq v \leq v^{+}_{limit})$ then $H(u,v) = 0$
else if $y_0 < -\frac{height}{2}p$
for v **do if** not $(-v^{-}_{limit} \leq v \leq -v^{+}_{limit})$ then $H(u,v) = 0$
//band-limited
$X(u,v) = \mathcal{F}\left[\widetilde{x}_l\right]$ // $\mathcal{F}$ denotes Fourier transform.
return $\mathcal{F}^{-1}\left[X(u,v) \times H(u,v)\right]$

Algorithm S2 Diffraction layer

Input: $\widetilde{x}$ // Wavefront. Data type is Complex.
Output: $\widetilde{x}'$ // Wavefront after phase modulation.
  Initialization:
    $\psi = \theta_0$
    // Learnable variable
  return $\widetilde{x} \times \mathrm{e}^{i\psi}$

Algorithm S3 Wavefront aberration added layer. (A bimodal Gaussian distribution is used.)

Input: $\widetilde{x}$,$z_4(x, y)$,$z_5(x, y)$,$z_6(x, y)$,μ,σ
    // z_4,z_5,z_6 are denotes Zernike olynomials corresponding to oblique astigmatism, defocus, and vertical astigmatism.
Output: $\widetilde{x}'$ // Aberration added wavefront.
for $i \leftarrow 4$ to 6 **do**
    $choice = rand.uniform(min = 0, max = 1)$ // A uniform random number of 0 or 1 is generated.
    $choice_{inverse} = 1 - choice$
    $coefficient_1 = rand.normal(mean = \mu, std = \sigma)$
    $coefficient_2 = rand.normal(mean = -\mu, std = \sigma)$
    $c_i = coefficient_1 \times choice + coefficient_2 \times choice_{inverse}$
end for
$\varphi = c_4 \times z_4(x, y) + c_5 \times z_5(x, y) + c_6 \times z_6(x, y)$
return $\widetilde{x} \times \mathrm{e}^{i\varphi}$

4.2 Random number generator

Figure S8 shows the generated random number and peak-to-valley distance (PV) value distributions. Aberration-adaptive learning uses random numbers to generate the coefficients of the Zernike polynomials to generate the various wavefront aberrations with PVs in a specific range. The PV range should be close to the PV of the wavefront aberrations overlapped on the system, which is often expected to be normally distributed. However, the general Gaussian distribution generates many values near zero when trying to generate equal positive and negative values. As a result, while the range of PVs is extended, the mode is close to zero, which prevents aberration-adaptive learning from working well in real space. To avoid this, and to generate positive and negative coefficients equally, we adopted the probability distribution shown in Fig. S8 (a) for generating the coefficients of the Zernike polynomials. The PV value distribution of the wavefront aberration generated from the probability distribution shown in Fig. S8 (a) is shown in Fig. S8 (b).

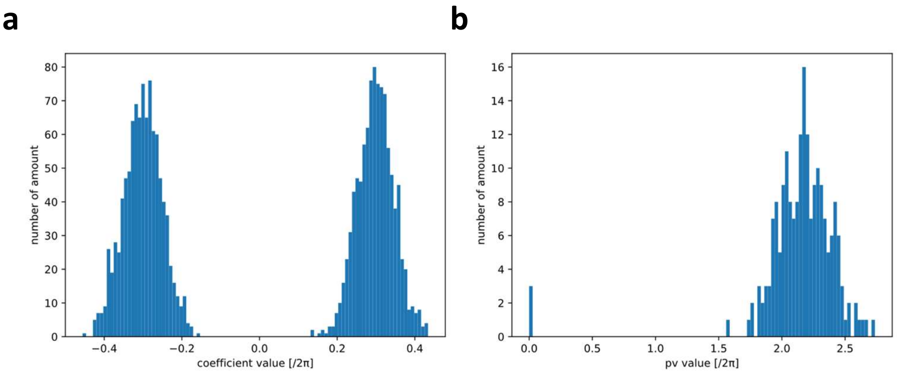

Fig. S8 (a) Probability distribution used in this study. The D^2NN constructed in this study needed to be adapted to the PV distribution of the wavefront aberration shown in (b). Therefore, we used the bimodal normal distribution to obtain the wavefront aberration distribution. (b) Distribution of PV values of wavefront generated from the probability distributions shown in (a) and Zernike polynomials.

In Fig. S8, the mean μ and standard deviation σ are set to $\pm 0.3 \times 2\pi$ and $0.05 \times 2\pi$, respectively, and these values are used in the experiment. These parameters were determined by performance comparison with some parameters added learning rate shown in Fig. S9. To determine the above parameters, we pre-selected and compared some candidates that would generate wavefront aberrations close to those measured experimentally by the wavefront sensor for the vHOEs fabricated. Specifically, we determined the parameters based on median accuracy and number of occurrences for each model. As shown in Fig. S9, the four models as follows have the highest median accuracy at 70% and the highest upper quartile accuracy at 74%: $[\mu, \sigma, learning\ rate] = [\pm 0.3 \times 2\pi, 0.05 \times 2\pi, 0.1]$, $[\pm 0.3 \times 2\pi, 0.05 \times 2\pi, 0.05]$, $[\pm 0.35 \times 2\pi, 0.1 \times 2\pi, 0.05]$, $[\pm 0.3 \times$

$2\pi, 0.05 \times 2\pi, 0.025]$. Among them, we determined the parameters used in our experiment to be $[\mu, \sigma, learning\ rate] = [\pm 0.3 \times 2\pi, 0.05 \times 2\pi, 0.1]$ since that model has the highest number of occurrences at 30 in the top quartile group of the box plot among the four models.

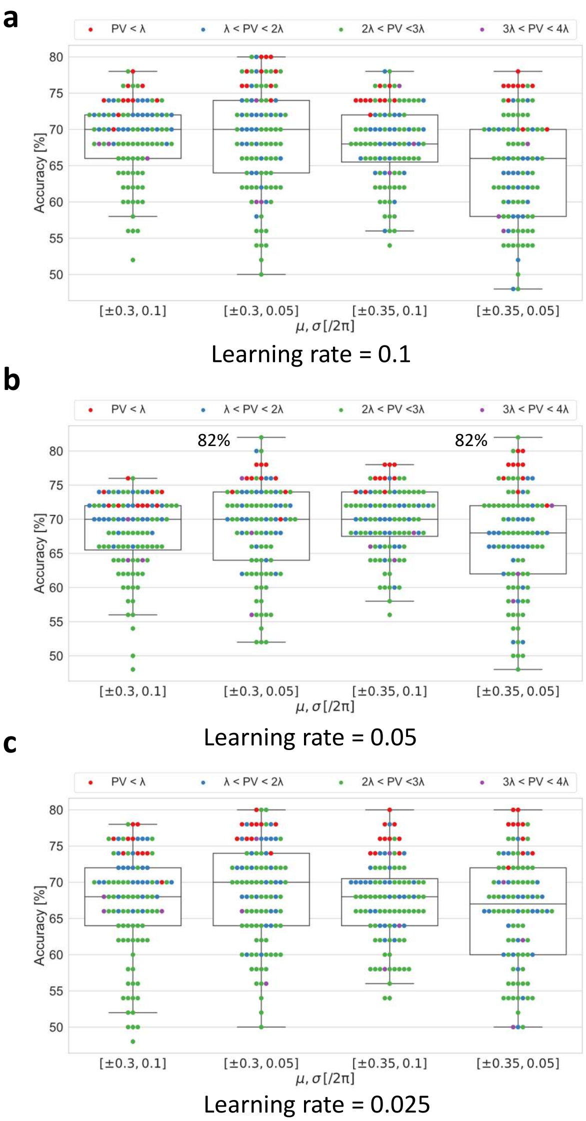

Fig. S9 Evaluation of each model with different parameters for learning rate, meanμ, and standard deviationσ: (a) learning rate 0.1, (b) learning rate 0.05, and (c) learning rate 0.025.

References

[1] Bruder, F.-K., Frank, J., Hansen, S., Künzel, R., Künzel, J., Lorenz, A., Manecke, C., Meisenheimer, R., Mills, J., Pitzer, L., *et al.*: Reliable photopolymer for new applications: vhoes recorded into bayfol hx film resisting the environment. In: Practical Holography XXXVI: Displays, Materials, and Applications, vol. 12026, p. 1202602 (2022). SPIE

[2] Matsushima, K.: Shifted angular spectrum method for off-axis numerical propagation. Optics Express **18**(17), 18453–18463 (2010)
